# Annotations

**Published:** 1906-03-31

**Authors:** 


### ANNOTATIONS.

### Devil-Driving.

There are not many varieties of treatment which cannot command a vogue in this country. Perhaps those who are weary of the attentions of the vibrator and electrical quack may be attracted by devil-driving as practised in the Malay States. The devil-driver described by Mr. T. C. Avetoom in the Journal of the Malaya Branch of the British Medical Association has a wonderful control over the muscles of his body and limbs, and can produce the most violent tremblings of the whole of his body when he begins to fight with the devil. The act of exorcism, as performed in the case of a boy with fever, is described as follows: "Having taken up his position before a table laden with food, he (the devil-driver) began by muttering to himself, one assistant ready to write down all that the devil would say through the old man, while the other started beating a gong. Presently a violent and furious trembling took hold of the devil-driver, and, while in this state, which lasted fully two minutes, he yelped and barked out short sentences, which were immediately written down on yellow paper with red ink by one assistant, while the other bravely continued beating the gong. Suddenly, and without any warning, the old man pounced on an old rusty sword which was also on the table, and taking it in his right hand and a handful of rice in his left, he ran up the stairs to the room where the lad lay. I of course followed. Having reached the lad he pelted him with the rice, and then, pulling out his own tongue, he ran the edge of the sword against it and started spitting what at first sight looked like blood all over the lad. The tremblings having gradually died off, the old man sank exhausted into a chair purposely placed for him. A little later I asked him to show me his tongue, but this he refused to do. I picked up the sword, and found that the cutting edge was fully an eighth of an inch in thickness. . . . This fearful and exhausting performance was done for the magnificent sum of 75 cents." Of course the thing could never become fashionable in this country at the absurdly low figure quoted in Penang; but with a minimum fee of five guineas the London society devil-driver would probably be a busy and prosperous person.

### Mr. Birrell and the Leicester Election.

Cabinet Ministers are not always courageous in their actions when a by-election is impending, and the course pursued by Mr. Birrell within a few days of the polling on Friday at Leicester merits recognition from those who consider that the welfare of the community is a matter of far greater importance than the success, or failure, of any particular candidate for Parliament. Of course, the Minister of Education, in refusing to remove the disabilities which prevent non-vaccinated persons from becoming pupil-teachers, school-masters or mistresses, ought to be able to feel that his decision commends itself to every sensible person. But in Leicester, at all events, there are many persons who are not sensible on the subject of vaccination, and when Mr. Birrell wrote his letter, he was perfectly well aware that to these it would give dire offence. As a matter of fact, he has been denounced by anti-vaccinationists in the town for sending "a wooden, mechanical, and unsympathetic" reply to the inquiry addressed to him by the local education authority. We think that he will bear this condemnation with equanimity. There may be room for a difference of opinion as to whether vaccination should be compulsory in the case of private individuals; there can be none in respect to individuals who receive the public money, and who, in the capacity of teachers, necessarily come in close contact with large numbers of children. If they are not prepared to observe the precaution against small-pox which the law of the land has laid down, there are other occupations open to them. We are glad that Mr. Birrell has not allowed the fear of the "conscientious objectors" who possess votes to hinder him from doing his duty as a member of the Imperial Government.

### How to Disinfect the Hands?

How is infection of operation wounds by the surgeon's hands to be avoided? Few problems seem easier at first sight than this one. From the time that the ill-fated Semmelweiss, by one of the grandest inspirations which have come to man, showed that the obstetrician could avoid carrying the contagion of puerperal fever on his hands by immersing them in chloride of lime, there has been the semblance of simplicity in the matter; and until the last few years there was a growing habit of attributing unexpected sepsis after an operation to anything but the true cause. As a matter of fact, until a short while ago bacteriological tests appeared to support the innocuousness of carefully "disinfected" hands. Now, however, the trend is all the other way. Haegler, Leedham Green and others have proved that previous bacteriological tests were fallacious, and that it is in truth impossible to render the hands completely sterile. Even hands which appear to be aseptic at the commencement of an operation, are now shown to give rise to copious growths of micro-organisms in nutrient media, if the test be applied when the operation is well under way. So that, in the light of present knowledge, the only method which approaches to absolute safety is for the surgeon to wear impervious gloves, which can be boiled before use. But, in spite of the assertions of certain surgeons who declare that they can operate better with than without gloves, these are of necessity a hindrance to manipulative dexterity, and we do not think that the method has come to stay. The moment a satisfactory and reliable method of disinfecting the skin is discovered, gloves will be discarded. The incongruous and needless refinements of the modern operation theatre merely teach surgeons and assistants how to bungle an operation when it has to be performed away from the kindly shelter of a hospital. Humanity would probably benefit if some of the money spent upon these refinements were devoted to a little organised research after a trustworthy and practical method of preventing infection of wounds by the surgeon's hands.

